# D-histidine exhibited anti-biofilm activity against *Aggregatibacter actinomycetemcomitans*

**DOI:** 10.1128/spectrum.01216-25

**Published:** 2025-09-29

**Authors:** Wenwen Shan, Fen Du, Haichuan Zhang, Jing Zhang, Xinyi Hu, Xinjiong Fan, Wuli Li

**Affiliations:** 1 Stomatological Hospital and College, Key Lab. of Oral Diseases Research of Anhui Province, Anhui Medical University 12485, Hefei, Anhui, China; 2 Faculty of Dentistry, The University of Hong Kong 71025 , Hong Kong SAR, China; 3 School of Basic Medical Sciences, Anhui Medical University 12485, Hefei, Anhui, China; College of New Jersey, Ewing, New Jersey, USA

**Keywords:** quorum sensing, periodontitis, biofilm, D-histidine, adhesion

## Abstract

*Aggregatibacter actinomycetemcomitans* is a key pathogen implicated in periodontitis. The bacterium in biofilms exhibits significant resistance to antimicrobial agents and host immune responses compared to its planktonic form, posing a major challenge for periodontal therapy. Recently, D-histidine has emerged as a promising anti-biofilm agent against *Pseudomonas aeruginosa* infections. However, its potential application in the oral field remains unexplored. This study investigated the anti-biofilm effect of D-histidine on *A. actinomycetemcomitans* and examined its influence on the expression of virulence factor genes to elucidate possible underlying mechanisms. Our results demonstrated that D-histidine inhibited biofilm formation and disrupted established biofilms in a concentration-dependent manner, without affecting bacterial growth. Furthermore, D-histidine downregulated the expression of virulence factors by inhibiting quorum sensing (QS)-related genes. Notably, combining D-histidine with antibiotics, such as amoxicillin, minocycline, and metronidazole, synergistically enhanced biofilm eradication and enabled the use of lower antibiotic dosages. These findings support the further evaluation of D-histidine as a potential anti-biofilm agent in the treatment of periodontitis.

**IMPORTANCE:**

The increasing prevalence of antibiotic-resistant *A. actinomycetemcomitans* biofilms posed a significant challenge in periodontitis management. This study demonstrated that D-histidine effectively targeted *A. actinomycetemcomitans* biofilms by disrupting structural integrity and suppressing virulence gene expression, without exerting bactericidal effects that could promote resistance development. Notably, D-histidine showed potent synergy with minocycline, significantly enhancing biofilm eradication while potentially enabling reduced antibiotic dosages. These findings established D-histidine as a promising adjunctive therapeutic agent, addressing the urgent need for novel approaches to overcome biofilm-associated antibiotic tolerance in periodontal treatment.

## INTRODUCTION

Periodontitis is a complex chronic inflammatory disease driven by interactions between microbial biofilm and the host immune response. It affects up to 60% of dentate adults worldwide, posing a significant public health burden (1). *Aggregatibacter actinomycetemcomitans* is one of the key Gram-negative pathogens associated with periodontitis (2). It possesses multiple virulence factors that facilitate adherence to tooth surfaces (3, 4). Furthermore, systemic inflammation triggered by *A. actinomycetemcomitans* and its toxins can exacerbate periodontal tissue damage, ultimately contributing to the destruction of connective tissue and bone.

Mechanical debridement remains the primary treatment strategies for periodontitis, whereas adjunctive therapies, such as systemic or local antibiotics, are considered supplementary approaches (5). However, their effectiveness is limited by the intrinsic resilience of biofilms and the growing threat of antibiotic resistance. The biofilm, characterized by microbial communities embedded within an extracellular polymeric substance (EPS) matrix, provides structural stability, facilitates aggregation, and confers protection from antimicrobial agents and host immune responses (6). As biofilms mature, the EPS matrix expands, enhancing resistance to clearance and promoting bacterial dispersal, thereby perpetuating infection cycles (7, 8). Prolonged or excessive use of antibiotics further exacerbates the risk of resistance development (9). Hence, there is an urgent need for alternative strategies capable of disrupting biofilms without contributing to antibiotic resistance.

Quorum sensing (QS) is a key regulatory system that regulates bacterial communication through the accumulation of autoinducer molecules in a cell density-dependent manner (10, 11). In *A. actinomycetemcomitans,* quorum sensing governs virulence expression and biofilm development primarily via the downstream two-component system qseBC (12–15). Targeting QS pathways, particularly two-component system qseBC, offers a promising strategy for inhibiting biofilm formation and reducing bacterial virulence without relying on conventional antibiotics (16).

Recent evidence suggests that certain amino acids can interfere with QS systems and modulate biofilm formation in various bacteria (17–19). Amino acids exist as two stereoisomers: L-amino acids (L-AAs), which predominate in mammalian proteins, and D-amino acids (D-AAs), which are rare in mammals but abundant in bacteria, where they play critical roles in peptidoglycan structure and function (20). Recent studies have highlighted the anti-biofilm and anti-virulence properties of specific D-AAs, such as D-tyrosine, which can attenuate QS signaling, reduce autoinducer molecule levels, and synergize with antibiotics (21, 22). However, the efficacy of D-AAs is highly dependent on the amino acid type and its underlying mechanism of action. Many bacteria naturally synthesize and secrete D-AAs, which inhibit biofilm formation in a variety of pathogenic species (17–19, 23, 24). For example, *Bacillus subtilis* produces D-leucine (D-Leu), D-methionine (D-Met), D-tyrosine (D-Tyr), and D-tryptophan (D-Trp) (23), and the inhibitory effects of D-Met/D-Trp on *Campylobacter jejuni* biofilms (17). Mechanistically, D-AAs can interfere with protein synthesis, hinder initial adhesion, and disrupt peptidoglycan integrity by competitively inhibiting peptidoglycan synthases, resulting in the incorporation of aberrant D-AAs into nascent cell wall material and subsequent loss of structural integrity (25–27). Notably, D-histidine has demonstrated potent anti-biofilm activity and antibiotic potentiation against pathogens such as *Pseudomonas aeruginosa* and *Porphyromonas gingivalis* (28, 29). Despite these promising findings, the potential effects of D-histidine on *A. actinomycetemcomitans* have not been fully studied. This study investigated the effects of D-histidine on biofilm formation of *A. actinomycetemcomitans, a*s well as its impact on the expression of adhesion factors and virulence-associated genes. We further explored the potential mechanisms underlying these effects, focusing on the QS system, and assessed the synergistic activity of D-histidine in combination with commonly used antibiotics, including amoxicillin, minocycline, and metronidazole. Our findings aim to demonstrate that D-histidine could be a novel anti-biofilm agent against *A. actinomycetemcomitans*, supporting its application for future clinical studies.

## MATERIALS AND METHODS

### Chemicals

D-histidine, amoxicillin, metronidazole, and minocycline were purchased from Sigma-Aldrich (St. Louis, MO, USA). Columbia blood agar plates, brain heart infusion broth, hemoglobin chloride, 0.02% vitamin K1 solution, and an oxygen indicator were purchased from Landbridge (Beijing, China). The DNA polymerase PrimeScrip RT Reagent Kit and TB Green Premix Ex Taq II and the RNeasy MinElute Cleanup Kit were purchased from SparkJade (Shandong, China). These chemicals and materials were used according to the manufacturer’s instructions. All other chemicals and reagents were of analytical grade and obtained from commercial sources unless otherwise stated.

### Bacterial strains

*A. actinomycetemcomitans* ATCC 43717 (Guangdong Microorganism Culture Collection Center, Guangzhou, China) was used as the representative bacterial strain. After the microorganism was grown on a Columbia blood agar plate, a single colony was extracted using an inoculation loop and cultured in Brain Heart Infusion Broth (Hopebio, Qingdao, China) containing 5 mg/L hemin and 1 mg/L vitamin K3 for 72 h in an anaerobic environment (85% N₂, 10% H₂, 5% CO₂).

### Bacterial growth

*A. actinomycetemcomitans* cultures were diluted to an optical density at 600 nm (OD_600_) of 0.1 in BHI medium according to a previously described protocol (30). The bacterial suspension was then added to 100 mM (final concentration) D-histidine and **v**ortex-mixed for homogenization. Control groups received equivalent volumes of sterile phosphate-buffered saline (PBS). The turbidity of the culture was measured at 600 nm at 4 h intervals.

### QS system, virulence factor, and adhesion genes analysis

*A. actinomycetemcomitans* cultures were diluted to an OD_600_ of 0.5 in BHI medium in a 12-well cell culture plate with 100 mM D-histidine. After incubation for 72 h at 37°C under static conditions, bacterial cells were harvested by centrifugation and immediately subjected to RNA extraction using the RNeasy MinElute Cleanup Kit. Real-time PCR (qPCR) was performed separately using the PrimeScript RT reagent kit. Primers for qPCR are listed in Table 1. RT-PCR was performed using the LightCycler 96 instrument with cycling conditions set at 95°C for 30 s, followed by 40 cycles of denaturation at 95°C for 5 s, and extension at 60°C for 30 s. The 16S rRNA gene served as the internal reference control, with primer sequences detailed in Table 1. A negative control containing RNase-free water instead of cDNA was included in each run. The 2^−ΔΔCt^ method was used to calculate the relative expression of the target gene against the internal reference gene (31), where ΔCt is the difference between the Ct value of the target gene minus that of the internal reference gene, and ΔΔCt is the difference between the ΔCt values across different samples.

**TABLE 1:** The qPCR primers

Gene	Primers (5′→3′)
16S ribosomal RNA (*16S rRNA*)	F: ACGCTGTAAACGGTGTCG R: TTGCATCGAATTAAACCACAT
Poly(glycerophosphate) α-glucosyltransferase (*pgA*)	F: GACGGTGATGCGGTATTGG R: GACCGATGATGGAGCTGAA
Cytolethal distending toxin subunit B (*cdtB*)	F: CAACAACACAATTCCAACCC R: GGCGATACCTGTCCATTCTT
Leukotoxin A (*ltxA*)	F: ATCAGCCCTTTGTCTTTCCTAG R: TGACCAAGTAAACTATCGCCG
Cytolethal distending toxin subunit A (*cdtA*)	F: GTCAACGAAGCTCCCAAGAACGCT R: TGTACCTCTCCTTAGATCCATCCT
Aggregative adherence fimbriae regulator (*aae*)	F: GGTTTTAGGCGGCACATTTA R: TGCTTGACCAACCATAACCA
*Escherichia coli* membrane antigen A (*emaA*)	F: CTGCAGCAACCGGGGATTAT R: AATGGATTGGTTGCCTTTAG
Fimbrial low-molecular-weight protein (*flp*)	F: TCAAAGCAATCGAAGCAATC R: GCAATAGCGATCAAACCGTA
Outer membrane protein 100 kDa (*omp100*)	F: ATCTTCAAGCCAAAACATC R: AAGGCTGCCGACATTAT
Quorum-sensing *Escherichia coli* regulator B (*qseB*)	F: GCAGTGGTGCTGGATTTAACCTTG R: GCGTTACTGCTCACTTCGTTATCCC
Quorum-sensing *Escherichia coli* sensor kinase C (*qseC*)	F: TAAGTGGAATAATTACAGCCTGCG R: TTGTTGTGCGTCAAACACTTGGTTC
Regulator of curli production A (*rcpA*)	F: GGGCATTAACTGGAGCCAC R: ATCCACCTCCGAAACCGAAG

### Biofilm formation

Following a previous study (32), a mid-exponential phase suspension of *A. actinomycetemcomitans* was diluted to OD_600_ = 0.5, and then 500 µL of the seed suspension was inoculated into 24-well cell culture plates containing round coverslips (14 mm diameter) at the bottom, followed by culture with 100 mM D-histidine. An equal volume of sterile ultrapure water was added to the control group. After 72 h of incubation at 37°C, planktonic cells were aspirated, and absorbance was measured at 600 nm to quantify planktonic bacteria. The plates were gently washed three times with phosphate-buffered saline (PBS). Biofilm formation was measured by crystal violet staining. For the crystal violet assay, each biofilm well was stained with a 0.1% crystal violet solution for 30 min. Subsequently, the microwells were rinsed three times with PBS and dried for 20 min, and the biofilm within them was dissolved in 95% ethanol; then the absorbance was measured at 590 nm to quantify biofilm formation and at 600 nm to quantify planktonic bacteria. The change in biofilm formation was calculated from the percentage change in absorbance (at 590 nm).

### Dispersal assay

A suspension of *A. actinomycetemcomitans* in the mid-exponential phase was diluted to OD_600_ = 0.5 (32), and 1 mL was seeded into 24-well cell culture plates containing round coverslips (14 mm diameter) at the bottom. After 72 h of cultivation at 37°C, the planktonic cells were aspirated, and the plates were gently washed three times with (PBS). The cells were then treated with 100 mM D-histidine and incubated for 24 h. Residual biofilms were detected using the methods described above. Changes in biofilm formation and in planktonic bacteria were calculated from the percentage change in absorbance.

### Biofilm staining and fluorescence microscopy

*A. actinomycetemcomitans* biofilms were cultured under static conditions (37°C, 72 h) on sterile glass coverslips in 24-well plates and cultured in BHI broth. After gentle rinsing with PBS to remove floating cells, the samples were fixed with 4% paraformaldehyde (15 min, RT), stained with 0.1% (wt/vol) aqueous crystal violet (20 min, RT), and washed rigorously with deionized water to remove unbound dye. Images were captured using a Zeiss Axio Observer 7 microscope (bright-field, exposure 200 ms). Z-stacks (1 µm step, 20 layers) from ≥9 random fields/coverslip were analyzed using threshold-based segmentation (Otsu method) in ImageJ. Biovolume (μm³/μm²) and surface coverage (%) were quantified.

### Statistical analysis

Statistical analyses were performed using SPSS version 24.0. Data are expressed as the mean ± standard deviation. Dunnett’s test was used to compare the test and control groups, and values of *P* < 0.05 were considered statistically significant. All experiments were performed in triplicate and repeated three times.

## RESULTS

### Effect of D-histidine on the growth of *A. actinomycetemcomitans*

**Fig 1:**
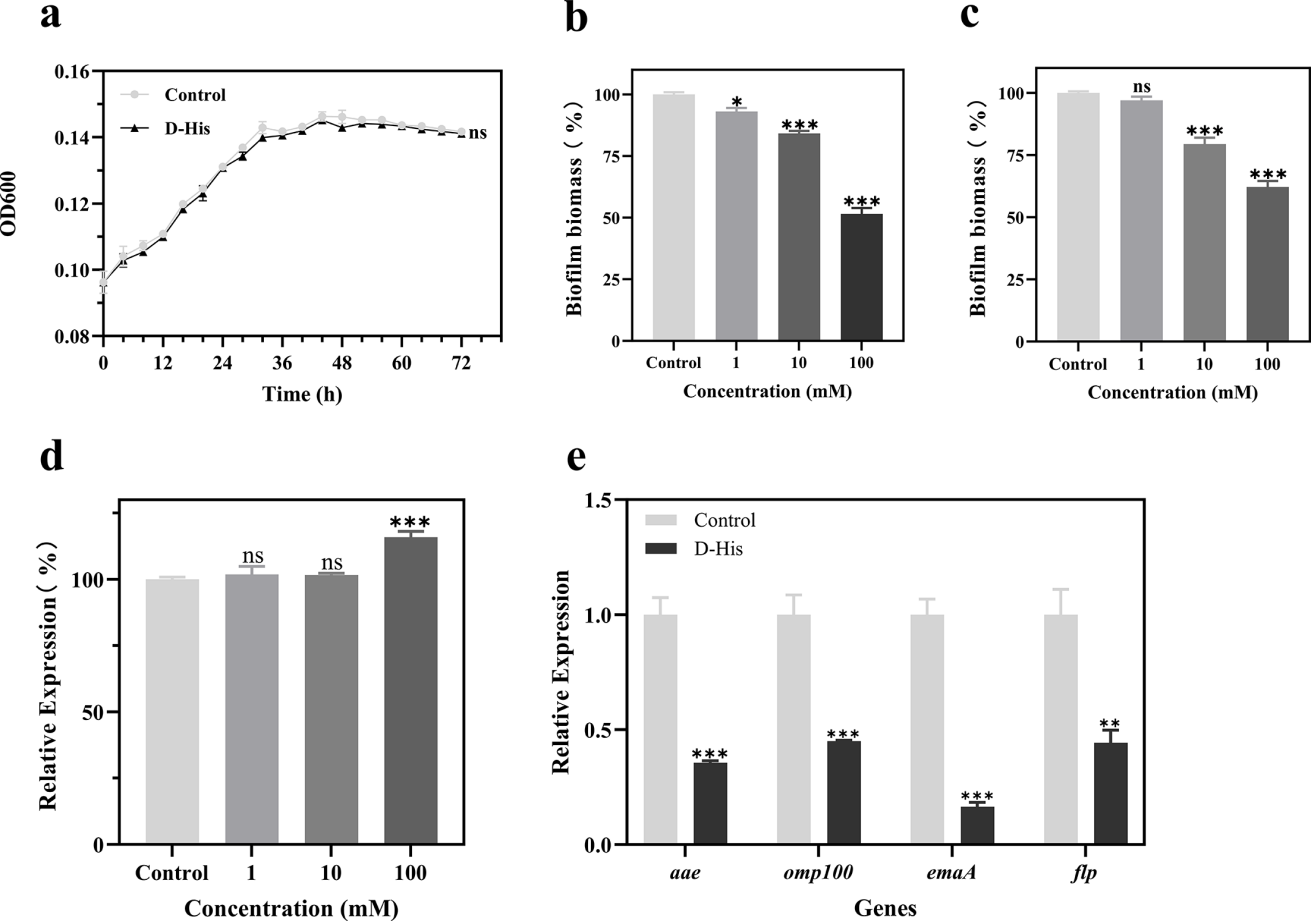
(**a**) Effect of D-histidine on the growth of *A. actinomycetemcomitans*. (**b**) Effect of D-histidine on biofilm formation. (**c**) Effect of D-histidine on pre-formed biofilm. (**d**) Detection of planktonic bacterial populations in biofilm formation with *A. actinomycetemcomitans*. (**e**) Effect of D-histidine on the expression of adhesion genes of *A. actinomycetemcomitans*. D-histidine: 100 mM. The data are shown as means ± the standard deviation (SD), *P < 0.05, **P < 0.01, ***P < 0.001 (vs control).

Growth curves (Fig. 1a) were plotted to determine the effect of D-histidine on the growth of *A. actinomycetemcomitans*. The growth curves indicated that the 100 mM concentration of D-histidine had no significant inhibitory or enhancing effect on the growth of *A. actinomycetemcomitans*. Subsequent studies used 100 mM and lower concentrations of D-histidine to investigate its potential effects on the QS system, virulence factors, adhesion genes, and biofilm formation of *A. actinomycetemcomitans*.

### Effect of D-histidine on biofilm formation and removal of formed biofilm of *A. actinomycetemcomitans*

We investigated the inhibitory and removal effects of D-histidine at concentrations of 0 mM, 1 mM, 10 mM, and 100 mM on the biofilms of *A. actinomycetemcomitans* using a crystal violet quantitative biofilm assay. Our results (Fig. 1b and c) showed that D-histidine could inhibit biofilm formation by *A. actinomycetemcomitans* and remove the formed biofilm in a concentration-dependent manner, with the best effects achieved at a D-histidine concentration of 100 mM, resulting in inhibition and removal rates of 49% and 38%, respectively (*P* < 0.001). Therefore, in the following experiments, we chose a concentration of 100 mM D-histidine in combination with different antibiotics to investigate its anti-biofilm effects.

### Effect of D-histidine on the adhesion of *A. actinomycetemcomitans*

The initial adhesion of bacteria is crucial for biofilm formation, and the adhesion of *A. actinomycetemcomitans* is increasingly being studied. We first investigated the number of bacterioplankton involved in the biofilm formation of *A. actinomycetemcomitans*. The results (Fig. 1d) showed that the amount of bacterioplankton in the presence of *A. actinomycetemcomitans* was significantly greater than in the control group under the intervention of 100 mM D-histidine (*P* < 0.001). Furthermore, D-histidine did not affect the growth of *A. actinomycetemcomitans*, leading us to speculate that D-histidine might inhibit the initial adhesion of *A. actinomycetemcomitans*, thereby inhibiting its biofilm formation. Therefore, we speculated that D-histidine might inhibit the initial adhesion of *A. actinomycetemcomitans* and, consequently, the formation of its biofilm. Subsequently, we performed RT-qPCR analysis on the genes that regulate the adhesion of *A. actinomycetemcomitans*. The results (Fig. 1e) showed that 100 mM D-histidine suppressed the expression of the *aae*, *omp100*, *emaA*, and *flp* genes (*P* < 0.05), with the downregulation of approximately 65%, 55%, 84%, and 56%, respectively, thereby inhibiting the initial adhesion of *A. actinomycetemcomitans*.

### Effect of D-histidine on virulence factors of *A. actinomycetemcomitans*

**Fig 2:**
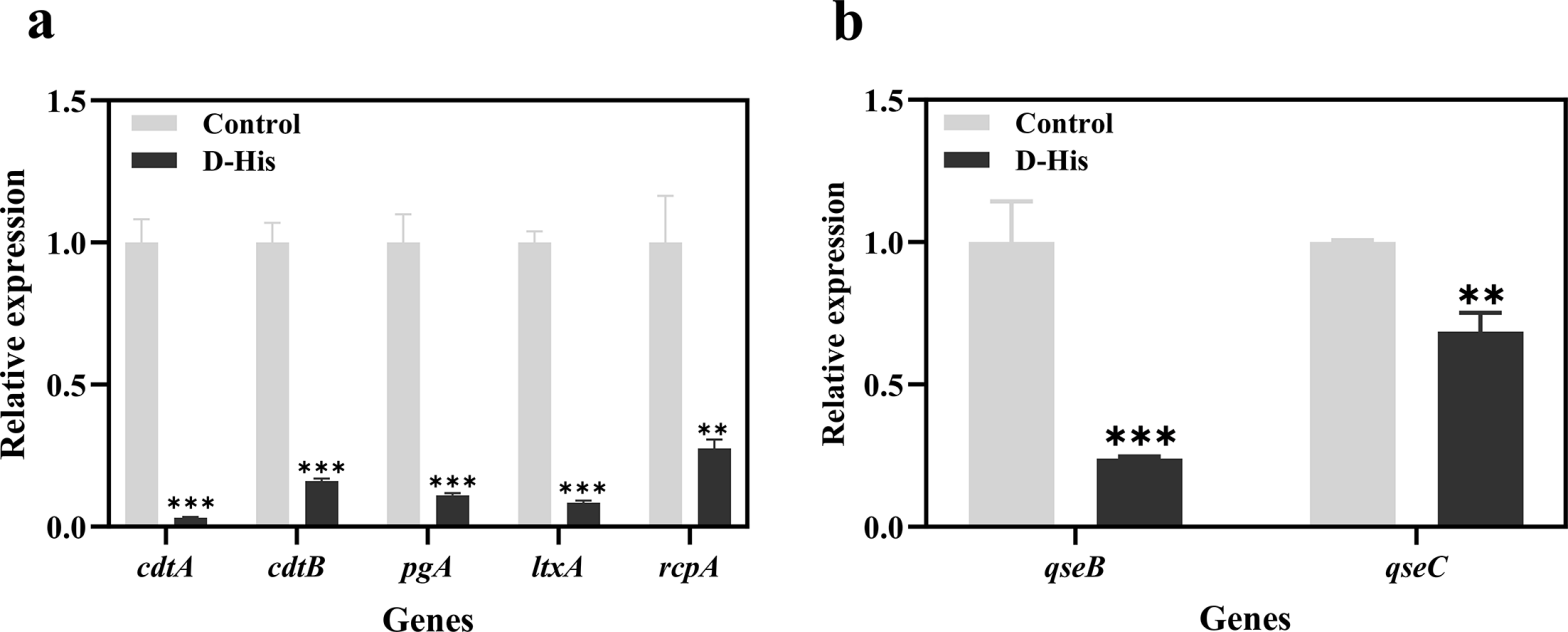
(**a**) Effect of D-histidine on the virulence gene expression of *A. actinomycetemcomitans* and (**b**) effect of D-histidine on the expression of QS genes in *A. actinomycetemcomitans*. D-histidine: 100 mM. The averages of the triplicate experiments represent each of the three experiments. The data are shown as means ± the standard deviation (SD), *P < 0.05, **P < 0.01, ***P < 0.001 (vs control).

*A. actinomycetemcomitans* is capable of producing many virulence factors, such as cytolethal distending toxin (*CDT*) and leukotoxin (*ITX*), which evade the body’s defense system and play a significant role in the early stage of periodontitis pathogenesis. We performed real-time quantitative PCR analysis of the genes regulating these virulence factors, and the results (Fig. 2a) showed that 100 mM D-histidine significantly inhibited the expression of the *cdtA*, *cdtB*, *pgA*, *itxA*, and *rcpA* genes (*P* < 0.05), with the downregulation of approximately 97%, 84%, 89%, 92%, and 73%, respectively.

### Effect of D-histidine on the expression of QS genes in *A. actinomycetemcomitans*

We investigated the effect of D-histidine on the transcript levels of the QS-related genes *qseB* and *qseC* of *A. actinomycetemcomitans* using real-time quantitative PCR experiments. The qseBC two-component system is associated with the QS of *A. actinomycetemcomitans* and is required for its adaptation and response to various environmental stimuli. It is also involved in the regulation of *A. actinomycetemcomitans* colonization, biofilm formation, and virulence gene expression. Our results (Fig. 2b) showed that the gene expression of both *qseB* and *qseC* was downregulated in *A. actinomycetemcomitans* following treatment with 100 mM D-histidine. Specifically, the expression of *qseC* was reduced by approximately 31% (*P* < 0.01), whereas the expression of *qseB* was more significantly affected, showing a reduction of approximately 76% (*P* < 0.001).

### Effect of D-histidine in combination with antibiotics on biofilm formation of *A. actinomycetemcomitans*

**Fig 3:**
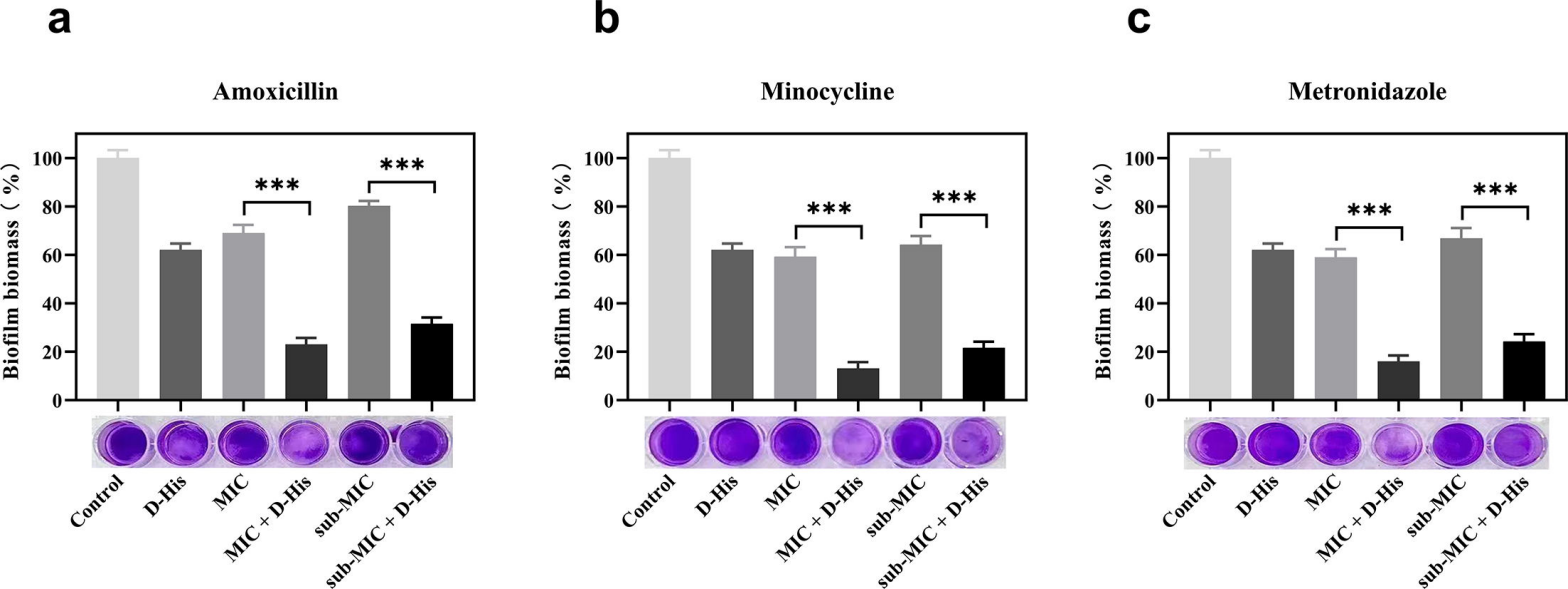
Effect of D-histidine in combination with antibiotics on pre-formed(mature/established) biofilm formation of *A. actinomycetemcomitans*. D-histidine:100 mM. (**a**) Amoxicillin, (**b**) minocycline, and (**c**) metronidazole. ***P < 0.001 (vs control).

**Fig 4:**
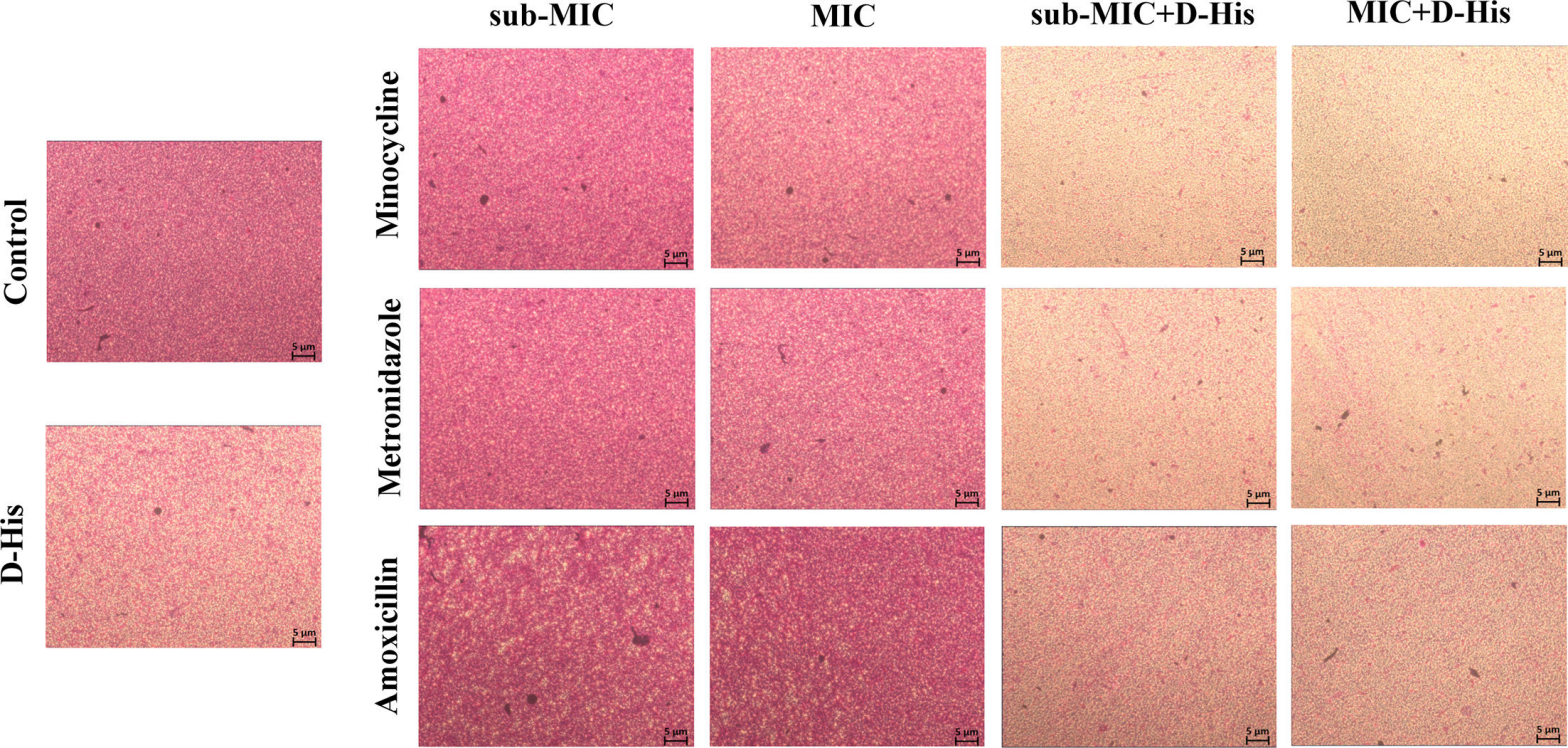
Microscopic analysis of the effect of 100 mM D-histidine in combination with antibiotics on biofilm formation of *A. actinomycetemcomitans*.

We evaluated the biofilm inhibitory effects of 100 mM D-histidine in combination with minocycline (MINO), metronidazole (MTZ), and amoxicillin (AMX) at both MIC and sub-MIC concentrations using a crystal violet quantitative biofilm assay (32). The MIC values for metronidazole and amoxicillin were 4 µg/mL, with a sub-MIC of 2 µg/mL, and for minocycline the MIC was 0.5 µg/mL, with a sub-MIC of 0.25 µg/mL, as determined by microdilution. Our results (Fig. 3) demonstrated that combinatorial treatment with D-histidine at MIC/sub-MIC concentrations significantly enhanced the biofilm-inhibitory efficacy of all three antibiotics relative to antibiotic monotherapy (*P* < 0.001*)*. This synergistic enhancement was morphologically corroborated by microscopic analysis of biofilm architecture (Fig. 4).

### Effect of D-histidine in combination with antibiotics on pre-formed biofilm of *A. actinomycetemcomitans*

**Fig 5:**
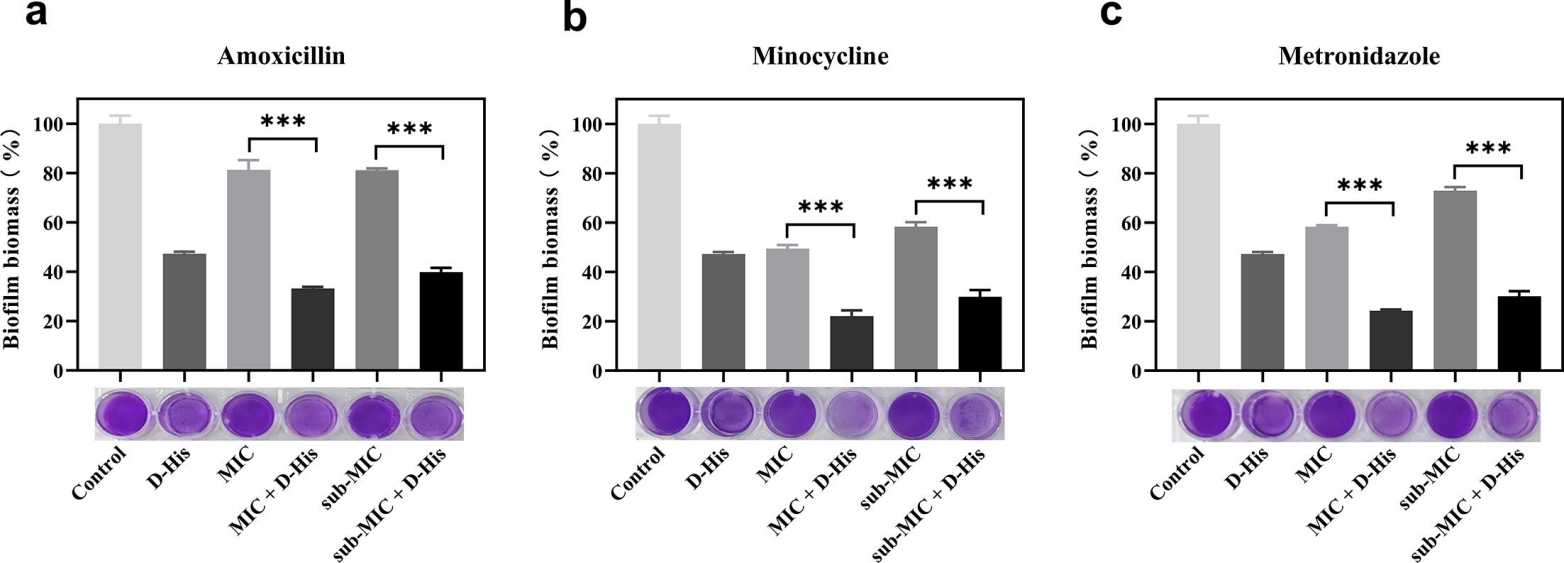
Effect of 100 mM D-histidine in combination with antibiotics on pre-formed biofilm of *A. actinomycetemcomitans*. D-histidine: 100 mM. (**a**) Amoxicillin, (**b**) minocycline, and (**c**) metronidazole. ***P < 0.001 (vs control).

**Fig 6:**
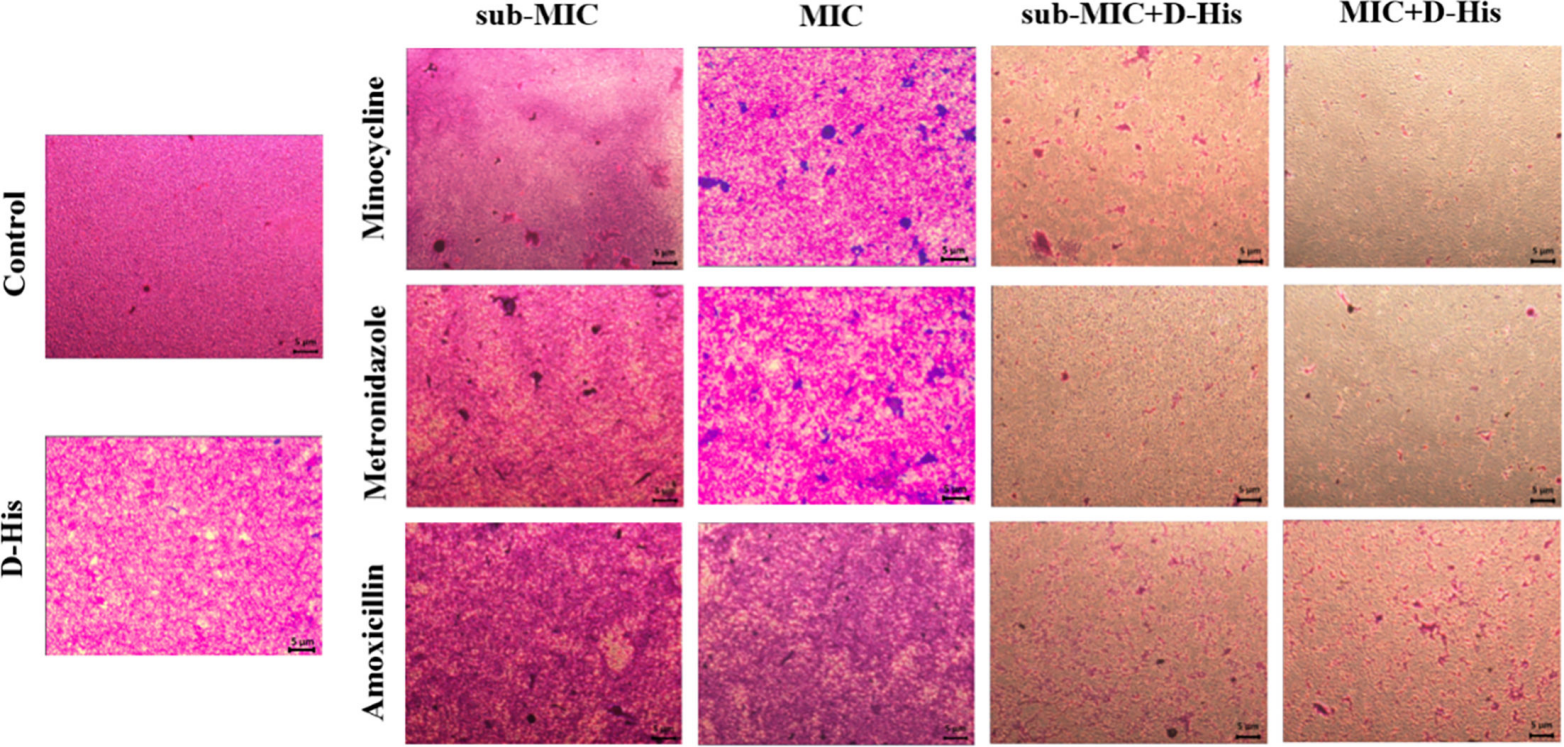
Microscopic analysis of the effect of 100 mM D-histidine in combination with antibiotics on pre-formed biofilm of *A. actinomycetemcomitans*.

We evaluated the effect of 100 mM D-histidine in combination with minocycline, metronidazole, and amoxicillin on pre-formed biofilms of *A. actinomycetemcomitans* at both MIC and sub-MIC concentrations using a crystal violet quantitative biofilm assay. Our results (Fig. 5 and 6) indicated that none of the antibiotics alone were effective in dispersing the biofilms formed by *A. actinomycetemcomitans*. However, the combination of all three antibiotics significantly enhanced biofilm dispersal at both MIC and sub-MIC concentrations when used with D-histidine (*P* < 0.001).

## DISCUSSION

Recent studies have raised concerns over the high prevalence of antibiotic resistance within the oral microbiota. *A. actinomycetemcomitans* isolates from subgingival samples of German volunteers exhibited high rates of resistance genes against multiple antibiotic classes, including beta-lactams, macrolides, nitroimidazoles, and tetracyclines (33, 34). In addition to genetic resistance, biofilms formed by these bacteria confer significant protection. Biofilms are structured communities encased within a protective matrix of Extracellular Polymeric Substances (EPS), primarily composed of water, proteins, polysaccharides, extracellular DNA (eDNA), and lipids (6). During biofilm development, EPS initially forms a sticky matrix enabling microbial attachment and aggregation (8). Subsequently, continuous EPS synthesis expands the matrix three-dimensionally, embedding cells into a networked core. Ultimately, this EPS-encased core establishes a biological scaffold driving mature 3D microcolony formation (7). This resilient EPS structure provides essential functions including adhesion, aggregation, structural stability, and crucially, protection. Consequently, the dense EPS matrix physically restricts antibiotic diffusion and chemically binds or degrades antimicrobial agents, further enhancing resistance (35). Therefore, conventional antibiotics such as minocycline and metronidazole exhibit reduced efficacy against biofilm-associated *A. actinomycetemcomitans*. These findings underscore the urgent need for novel strategies that can disrupt biofilms and enhance antibiotic effectiveness.

D-amino acids (D-AAs) have emerged as promising candidates for combating biofilm-associated infections. While studies have shown that D-AA mixtures containing D-histidine can inhibit biofilm proliferation in some species, such as *P. gingivalis* (29), their effects appear to be species-specific. In our study, D-histidine did not impact the growth of *A. actinomycetemcomitans*, suggesting its antibiofilm activity is not due to bactericidal effects, but rather to interference with processes critical for biofilm formation.

### D-Histidine targets genes involved in adhesion in *A. actinomycetemcomitans* biofilms

Our results demonstrated that D-histidine inhibits biofilm formation and promotes dispersal of established *A. actinomycetemcomitans* biofilms in a dose-dependent manner, with the highest effect observed at 100 mM. Importantly, D-histidine increased the number of planktonic bacteria without affecting overall bacterial growth, indicating that its primary mechanism involves disruption of initial bacterial adhesion. This is supported by the significant downregulation of genes encoding key adhesive structures, including Flp pili and non-pilus adhesins such as *EmaA*, *Aae*, and *Omp100* (3, 36–41), following D-histidine treatment. Specifically, Flp pili suppression abolishes bacterial aggregation, which is essential for microcolony formation and subsequent EPS matrix nucleation (42, 43). These adhesins are crucial for bacterial attachment to surfaces and host cells, and their inhibition likely accounts for the observed reduction in biofilm biomass.

### D-Histidine reduces virulence factor expression via inhibition of quorum sensing system

Beyond adhesion, virulence factors play an essential role in biofilm formation and pathogenicity of *A. actinomycetemcomitans*. Cytolethal distending toxin *(CDT)* and leukotoxin A *(LtxA)* are two primary exotoxins in *A. actinomycetemcomitans. CDT*, consisting of three subunits-—*CdtA, CdtB*, and *CdtC*, is a genotoxin that disrupts the host cell cycle, causing cell death and tissue destruction (44, 45). *LtxA* is a major virulence factor that targets and kills leukocytes (3, 44). Crucially, biofilm maturation depends on extracellular polymeric substances (EPS). Unlike many bacteria, *A. actinomycetemcomitans* relies exclusively on poly-N-acetylglucosaminoglycan *(PGA)* as its dominant EPS. Loss of *PGA* function impairs colonization efficacy, reduces osteolytic activity, and suppresses virulence gene expression (46, 47). *PGA* forms a protective matrix embedding bacterial cells and virulence factors (e.g., *CDT, LtxA*), conferring structural stability and resistance absent in vulnerable planktonic cells. Our study found that D-histidine treatment significantly downregulated the expression of major virulence genes, including those encoding cytolethal distending toxin *(CDT)*, leukotoxin A *(LtxA)*, the rough colony protein *RcpA*, and poly-N-acetylglucosaminoglycan *(PGA)*. The reduction in these factors is likely to decrease bacterial adhesion, biofilm stability, and pathogenicity, potentially rendering the bacteria more susceptible to antibiotics and host defenses.

The expression of virulence factors and adhesins is regulated, in part, by QS systems, including the qseBC two-component system. QseBC functions as a membrane protein responsible for sensing extracellular signals (48). QseBC functions as a membrane protein responsible for sensing extracellular signals (48). Upon ligand binding, it initiates a phosphorylation cascade that activates virulence gene expression (e.g., toxin secretion) and modulates bacterial behaviors (e.g., motility), ultimately enhancing pathogenicity (49). Weigel et al. also reported that *qseBC* can influence both biofilm formation and virulence production in *A. actinomycetemcomitans* (50). Additionally, Novak et al. (12) showed that *qseBC* is required to stimulate biofilm formation of *A. actinomycetemcomitans* in an animal model of periodontitis. Our data showed that D-histidine significantly downregulated the expression of *qseB* and *qseC*, suggesting that D-histidine disrupts mature biofilm and reduces the pathogenicity by suppressing the QS system pathway.

### D-Histidine strengths antibiotic efficacy against *A. actinomycetemcomitans* biofilm

Importantly, we found that combining D-histidine with antibiotics enhanced the inhibitory and clearance effects on *A. actinomycetemcomitans* biofilms compared to antibiotics treatment alone. The combination of D-histidine with minocycline, in particular, exhibited a pronounced synergistic effect at both MIC and sub-MIC concentrations. This enhanced efficacy may be attributed to the complementary mechanisms of action: D-histidine disrupts bacterial adhesion and reduces the expression of virulence factors, while antibiotics target intracellular processes. Notably, this synergy exceeded that observed with D-histidine/amoxicillin, reflecting amoxicillin’s vulnerability to critical EPS-mediated resistance mechanisms. Specifically, the extracellular matrix impedes amoxicillin penetration while simultaneously subjecting the antibiotic to enzymatic degradation by β-lactamases, which constitute key EPS protein components that hydrolyze β-lactams, and facilitates non-specific binding (51). Minocycline’s lipophilicity (52) enables superior biofilm penetration and protein synthesis inhibition (53), whereas amoxicillin’s efficacy against planktonic bacteria. In contrast, amoxicillin, though potent against planktonic bacteria (54), is compromised in biofilms by β-lactamase degradation and EPS penetration barriers (55). As a result, the synergy between D-histidine and amoxicillin is relatively weak, reflecting the inherent limitations of amoxicillin in biofilm-associated infections.

### Conclusion

In summary, our study demonstrates that D-histidine effectively inhibits biofilm formation by *A. actinomycetemcomitans* through suppression of adhesion, virulence factor production, and QS-related gene expression. When combined with antibiotics, D-histidine enhances antimicrobial efficacy against biofilms, suggesting a promising strategy to improve treatment outcomes and mitigate the emergence of antibiotic resistance in periodontal disease. Further research is warranted to elucidate the precise mechanisms of action and assess the clinical potential and safety of D-histidine as an adjunctive therapy in periodontitis management.

## Data Availability

Data used in the present study are available from the corresponding author on reasonable request.
